# How to Evaluate Adenomyosis in Patients Affected by Endometriosis?

**DOI:** 10.1155/2014/507230

**Published:** 2014-08-12

**Authors:** Nadine Di Donato, Renato Seracchioli

**Affiliations:** Minimally Invasive Gynaecological Surgery Unit, S. Orsola-Malpighi Hospital, University of Bologna, Via Massarenti 13, 40138 Bologna, Italy

## Abstract

*Objective*. The aim of the study is to evaluate adenomyosis in patients undergoing surgery for different type of endometriosis. It is an observational study including women with preoperative ultrasound diagnosis of adenomyosis. Demographic data and symptoms were recorded (age, body mass index, parity, history of previous surgery, dysmenorrhea, dyspareunia, dyschezia, dysuria, and abnormal uterine bleeding). Moreover a particular endometrial shape “question mark sign” linked to the presence of adenomyosis was assessed.* Results*. From 217 patients with ultrasound diagnosis of adenomyosis, we found 73 with ovarian histological confirmation of endometriosis, 92 with deep infiltrating endometriosis, and 52 patients who underwent surgery for infertility. Women with adenomyosis alone represented the oldest group of patients (37.8 ± 5.18 years, *P* = 0.02). Deep endometriosis patients were nulliparous more frequently (*P* < 0.0001), had history of previous surgery (*P* = 0.004), and complained of more intense pain symptoms than other groups. Adenomyosis alone was significantly associated with abnormal uterine bleeding (*P* < 0.0001). The question mark sign was found to be strongly related to posterior deep infiltrating endometriosis (*P* = 0.01).* Conclusion*. Our study confirmed the strong relationship between adenomyosis and endometriosis and evaluated demographic aspects and symptoms in patients affected by different type of endometriosis.

## 1. Introduction

Adenomyosis is a benign condition of the uterus, defined by the presence of endometrial glands and stroma within the myometrium. It is known as a histological diagnosis but it has a clinical dignity showing symptoms (dysmenorrhea, dyspareunia, abnormal uterine bleeding, and infertility) and sharing some pathogenic mechanisms with endometriosis [1]. Most of the major authors of the first half of the past century dealing with the disease considered pelvic endometriosis and uterine adenomyosis as variants of the same disease process [2, 3]. Also, Sampson (1927), although focusing mainly on the aetiology of the pelvic dissemination of the disease, mentioned uterine adenomyosis and referred to it as “primary endometriosis” [4]. Bazot and colleagues reported that 27% of women with endometriosis had concomitant adenomyosis [5]. Moreover, a 42.76% prevalence of adenomyosis in patients with endometriosis has been recently identified in patients reporting severe or incapacitating dysmenorrhea and deep dyspareunia and in patients with endometriosis of the rectosigmoid [6]. A common pathogenesis for adenomyosis and endometriosis has been hypothesized [7–9] and it was argued that endometrial stroma being in direct contact with the underlying myometrium allows communication and interaction, thus facilitating endometrial invagination or invasion of a structurally weakened myometrium during periods of regeneration, healing, and reepithelization [10]. Mechanical damage [11] to and/or physical disruption of the endometrial-myometrial interface may be due to dysfunctional uterine hyperperistalsis and/or dysfunctional contractility of the subendometrial myometrium.

Finally, considering adenomyosis as consequence of infiltration of basal endometrium into the underlying myometrium [8, 12], a correlation between the stage of endometriosis and the depth of adenomyotic infiltration has been reported [13]. Up to the present, in literature there is a lack of studies evaluating specifically demographic characteristics and symptoms in patients with adenomyosis and different type of endometriosis. In this view, we considered in our paper women with diagnosis of adenomyosis and endometriosis undergoing surgery and we recorded their demographic and clinical characteristics.

## 2. Materials and Methods

### 2.1. Design and Patients

For this observational study, ethical approval was obtained from the local ethics committee (198/2013/O/OssN). From January 2010 to December 2012, in the Minimally Invasive Gynecological Surgery Unit of the Department of Gynecology, S. Orsola-Malpighi Hospital, University of Bologna, women with ultrasound diagnosis of adenomyosis and endometriosis undergoing surgery for endometriosis and patients with ultrasound diagnosis of adenomyosis undergoing surgery for infertility were enrolled. Patients were divided according to the type of endometriosis (superficial, ovarian, and deep infiltrating endometriosis). This anatomic classification based on depth of infiltration of endometriotic nodules has been largely accepted by many authors [14, 15]. Deep infiltrating endometriosis was considered as a particular form of endometriosis that penetrates >5 mm under the peritoneal surface [15]. In case more than one condition was found, we considered the more advanced disease to classify women in the three different types of endometriosis. In endometriosis groups, histological confirmation of endometriosis was considered as an inclusion criterion. We excluded women who had previously undergone a hysterectomy, those who were pregnant women, those with malignant gynaecological disease, and those who received hormonal therapy (gonadotropin-releasing hormone agonist, levonorgestrel intrauterine system) in the preceding 3 months before surgery.

The following demographic data and symptoms were recorded: age, body mass index (BMI), history of previous surgery, dysmenorrhea, dyspareunia, dyschezia, dysuria, and abnormal uterine bleeding. The level of patient's discomfort and pain was evaluated by the visual analog scale (VAS) system, utilizing a 10 cm line with the extreme points 0 and 10 corresponding to “no pain” and “maximum pain,” respectively. Menstrual bleeding was assessed with the use of the pictorial blood loss assessment chart (PBAC), [16] a validated method used to objectively estimate blood loss; monthly scores range from 0 to more than 500, with higher numbers indicating more bleeding, and menorrhagia was defined as a PBAC > 100 during 1 menstrual period, which corresponds to a blood loss of more than 80 mL.

A transvaginal ultrasound scan was then done before surgery by a single operator (G.M.) and other diagnostic tests, such as magnetic resonance imaging (MRI) and computed tomography (CT), were performed when indicated to evaluate the presence, the localization, and the extension of endometriosis lesions. Sonographic examinations were performed in a systematic manner by a single sonographer, using high-quality ultrasound machine (Voluson S8, GE Milwaukee, WI, USA) equipped with a transvaginal wide-band 5.0–9.0 MHz transducer, which ensured a consistent approach to data collection and ultrasound examination. First, the uterus was examined in the longitudinal plane to identify the endometrium. The probe was then rotated 90° anticlockwise and cervical canal and the uterine cavity were visualized in the transverse plane. The myometrium was systematically examined for the sonographic features associated with adenomyosis. Diagnosis of adenomyosis was made when 3 or more of the following sonographic features were present: heterogeneous myometrial echotexture (presence of an indistinctly myometrial area with decreased or increased echogenicity), globular-appearing uterus (regular enlarged uterus), asymmetrical thickness of anteroposterior wall of the myometrium, subendometrial myometrial cysts (round anechoic areas of 1–7 mm diameter), subendometrial echogenic linear striations (radiate pattern of thin acoustic shadowing not arising from echogenic foci), or poor definition of the endometrial-myometrial junction, according to previous studies [17–19].

**Figure 1:**
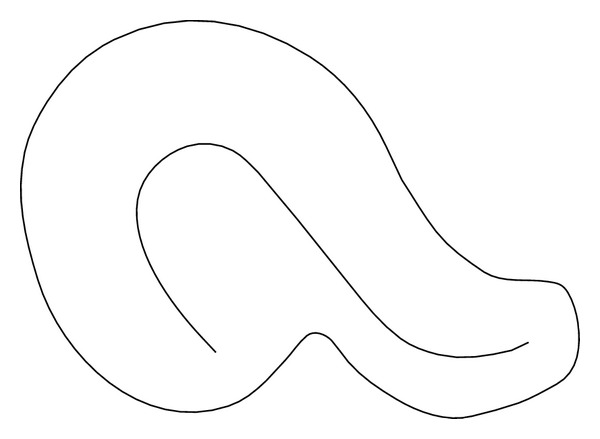
Question mark sign.

**Figure 2:**
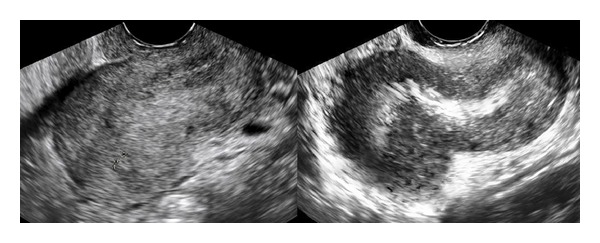
Ultrasound image of question mark sign.

Moreover, ultrasound diagnosis of endometriosis was made when ovarian endometriomas or endometriotic nodules were visualized. Ovarian cysts were classified as endometriomas when they appeared as well-circumscribed thick-walled cysts that contained homogeneous low-level internal echoes (“ground glass”). Endometriotic nodules were typically visualized as stellate hypoechoic or isoechogenic solid masses with irregular outer margins which were tender on palpation and fixed to the surrounding pelvic structures, as previously described [20–23]. The diagnosis of DIE was made if at least 1 structure in the anterior or posterior compartment showed the presence of retroperitoneal abnormal hypoechoic linear or nodular thickening with irregular contours and no vascular Doppler signals, as described previously and validated ultrasonographic criteria [24, 25]. Locations for DIE in the anterior (bladder) or posterolateral compartment (vagina, rectovaginal septum, torus and uterosacral ligaments, parametria and ureteral involvement, rectum, and rectosigmoid junction) were examined. Concerning ultrasound adenomyosis evaluation, we described and recorded an additional finding which has been observed in our clinical practice. In adenomyosis cases, we identified a particular endometrial shape named* question mark sign (“?”)*. The* “?” sign* was considered positive when the endometrium of the uterine fundus was deviated versus the pelvis posterior compartment (Figures 1 and 2).

### 2.2. Statistical Analysis

We reported means, standard deviations and medians for continuous variables, and frequency counts and percentages for nominal or categorical variables. To assess differences between groups of women, Fisher's exact test generalized for more than two groups was performed for nominal and categorical variables. Continuous data were analyzed with one-way ANOVA and Tukey's post hoc pair wise comparisons for continuous variables. An effect was deemed statistically significant at 0.05. All the analysis was performed with the software SPSS 11.0 software (SPSS Inc., Chicago, IL, USA).

## 3. Results

Demographic and clinical characteristics of women included in the study are summarized in Table 1.

A total of 268 women were enrolled. Of the 268 women, 51 were excluded from the data analysis as 21 patients had no histological diagnosis of endometriosis, 26 had intolerance to transvaginal ultrasound examination, and 4 had previously undergone a hysterectomy.

From 217 patients included in the study with ultrasound diagnosis of adenomyosis, we found 66 with ovarian histological confirmation of endometriosis, 92 with DIE, and 52 patients who underwent surgery for infertility. Only seven women were found with superficial endometriosis alone. For the analysis of data, we considered them together with the ovarian group. Nine women during laparoscopic surgery for endometriosis underwent hysterectomy. For all of them adenomyosis diagnosis was histologically confirmed.

Women with adenomyosis alone represented the oldest group of patients (37.8 ± 5.18 years, *P* = 0.02); instead, BMI did not differ between groups. Concerning the characteristics recorded, we found that patients with DIE were nulliparous more frequently than other groups (*P* < 0.0001) and comparing to endometriosis patients (87/92, 94.6% DIE; 54/73, 73.9% ovarian endometriosis), only 27 (27/52 51.9%) women with adenomyosis alone were nulliparous. Moreover, in women with one or more births adenomyosis alone was significantly more frequent than in the others (*P* < 0.0001).

We evaluated the history of previous surgical procedures and we found that women with adenomyosis and DIE had more frequently a history of previous surgery (*P* = 0.004). Regarding symptoms, DIE group complained of more intense pain symptoms (dysmenorrhea, chronic pelvic pain, dyspareunia, dysuria, and dyschezia) than other groups. Moreover, patients with only adenomyosis were more likely to have abnormal uterine bleeding (*P* < 0.0001) (Table 2).

Describing the particular endometrial shape mentioned above, we found a strong relationship between this sonographic sign and adenomyosis associated deep infiltrating endometriosis. It was significantly (*P* < 0.0001) more present in DIE group (34/92 [37%]) than in the others (ovarian 7/73 [9.6%] and adenomyosis alone 4/52 [7.7%]). Specifically, evaluating the different localizations of DIE (anterior and posterior), we reported that it was significantly (*P* = 0.01) associated with posterior deep nodule (23/46 [50%] posterior DIE versus 11/46 [23.9%] anterior DIE).

## 4. Discussion

Today, several authors have described the association between adenomyosis and endometriosis, particularly DIE [26–28]. In a prospective study Kissler et al. found that severe dysmenorrhea of long duration in patients with endometriosis is significantly related to uterine adenomyosis [27]. Moreover, Larsen et al. found a correlation between the severity of endometriosis and the degree of uterine adenomyosis and Gonzalez et al. found again a correlation between uterine adenomyosis and deep endometriosis with poor prognosis, particularly endometriosis of the rectosigmoid [6]. In our study, we evaluated clinical and demographic characteristics related to adenomyosis in patients undergoing surgery for different type of endometriosis. We identified a particular endometrial shape associated with adenomyosis and we named this specific endometrial finding “*question mark sign*.” The endometrium was deviated versus the pelvis posterior compartment from the fundus to the cervix. Moreover, we reported that the question mark sign was strongly related to the presence of posterior deep infiltrating endometriosis.

Therefore, it is interesting to look for this endometrial sign during the preoperative ultrasound evaluation in the way to facilitate the diagnosis of both posterior deep infiltrating endometriosis and adenomyosis. Probably, the uterus is deviated by the nodule in the posterior compartment of the pelvis changing the normal endometrial shape. Commonly, endometriosis nodules have the capacity to attract structures around the pelvis. Moreover, from literature, the posterior wall of the uterus was predominantly affected by adenomyosis [29, 30]. However, no data are available that show an increased mechanical stress of the posterior uterine wall due to chronic uterine peristalsis and hyperperistalsis and a relationship of the site of predilection of adenomyosis with ante- or retroflection of the uterus [29, 30]. There is indirect evidence of an archimetral hyperestrogenism in women with endometriosis that interferes with the ovarian control of uterine peristaltic activity resulting in uterine hyperperistalsis [31, 32]. Moreover, it was argued that endometrial stroma being in direct contact with the underlying myometrium allows communication and interaction, thus facilitating endometrial invagination or invasion of a structurally weakened myometrium during periods of regeneration, healing, and reepithelization [10, 11].

From our results, we found that patients with DIE and adenomyosis were nulliparous more frequently and complained of more intense pain symptoms (dysmenorrhea, chronic pelvic pain, dyspareunia, dysuria, and dyschezia) than other groups with the exception of abnormal uterine bleeding which was complained by the majority of women with only adenomyosis. Heavy bleeding may be positively related to the depth of penetration of individual adenomyotic glands into the myometrium and to the density of deep endometrial glands within the myometrium [33].

In concordance with previous clinical evidence, we found that a high percentage of women with adenomyosis were multiparous [11, 18, 34, 35]. Instead, patients affected by endometriosis are frequently associated with infertility because of anatomical and immunological alterations caused by endometriosis. Generally, endometriosis is common in women with subfertility and can affect fertility at different level, from the induction of a local inflammation and decrease in endometrial receptivity to mechanical obstruction and altered sexual function [36].

There are some potential mechanisms associated with parity and adenomyosis pathology. First, pregnancy might facilitate formation of adenomyosis by allowing adenomyotic foci to be included in the myometrium due to the invasive nature of the trophoblast on the extension of myometrial fibers. Second, the possibility of Cesarean section may lead to iatrogenic adenomyosis [11, 37]. Third, the hormonal milieu of pregnancy may favour the development of islands of ectopic endometrium [38]. A recent review in 2013 defined a number of factors that encourage the development of adenomyosis (spontaneous miscarriage, curettage, hysteroscopic resection of the endometrium, uterine myomectomy, caesarean section, and taking Tamoxifen) and underlined that the main factor is having had more than one pregnancy [39]. In agreement with our data, Shrestha found that the frequency of adenomyosis was higher in parous women in comparison with nulliparous [40]. Moreover, Vercellini and colleagues suggested that parity may be associated with an increased frequency of adenomyosis [38]. Other studies reported the same relationship between parity and adenomyosis [34, 41].

Concerning symptoms, DIE is the most severe form of endometriosis, associated with infertility or pain symptoms, including chronic pelvic pain, dysmenorrhea, dyspareunia, dysuria, and dyschezia [42]. Nowadays, it is clarified that there is an overlap in the symptom complexes of both endometriosis and adenomyosis. In endometriosis, the presence of adenomyosis is found to be a risk factor for dysmenorrhea severity [6]. As described before, dysmenorrhea increases with greater depth of penetration of the adenomyotic process and to the density of deep endometrial glands into myometrium [43]; instead dyspareunia and chronic pain are not considered constant symptoms in patients with adenomyosis [5, 44]. Lazzeri and colleagues showed a persistence of dyspareunia in women after surgery for DIE when adenomyosis was present [45]. Another study reported pain recurrence in DIE, despite the radicality of the surgery [46]. Consequently, a correct counselling about pain recurrence is required in case of coexistence of both pathological entities. Surgery for posterior DIE with coexistence of adenomyosis could be not resolved for postoperative pain relief because of adenomyosis persistence. This information allows the chance of a correct counselling before surgery and should help to develop more effective treatment strategies in women affected by DIE and adenomyosis.

The strength of our study is the large number of women with endometriosis histologically confirmed and that it was conducted in a tertiary care university hospital, by clinicians highly skilled in endometriosis management. Moreover, sonographic examinations were performed in a systematic manner by a single sonographer, using high-quality ultrasound machine. The present study has also major limitations. First, the lack of histological confirmation of adenomyosis for the majority of patients included. However, recently transvaginal ultrasound has reached a high level of accuracy and many authors have reported the high agreement between ultrasound diagnosis of adenomyosis and histological findings [17, 47]. A recent review [48] reported that transvaginal ultrasound should be the primary tool for the diagnosis of adenomyosis, with MRI being used when transvaginal ultrasound is inconclusive or when large fibroids are present. Secondly, being patients included in the study with a mean age of 35 years old, it can be a possible bias for evaluation of the natural history of adenomyosis pathology. Since the adenomyotic nodules communicate with the uterine cavity, pathophysiologically a continuous process from initial to deep infiltration must exist. Authors reported that endometriosis-associated adenomyosis progresses with age [13, 49].

In conclusion, our data confirmed the strong relationship between adenomyosis and endometriosis and evaluated demographic aspects and symptoms in patients affected by different type of endometriosis. Our findings suggest that adenomyosis and endometriosis share same symptoms with important difference linked to the type of endometriosis and help to better understand the endometriosis-adenomyosis relationship and their associated factors.

## Figures and Tables

**Table 1:** Demographic and clinical characteristics of all women included.

Variables	*n* = 217
Age (y) (mean ± SD)	35.3 ± 5.88
BMI (body mass index kg/m^2^) (mean ± SD)	22.9 ± 3.57
Parity (*N* [%])	
0	168 (77.4%)
≥1	49 (22.6%)
Endometriosis (*N* [%])	
Adenomyosis alone	52 (24%)
Ovarian	73 (33.6%)
DIE	92 (42.4%)
Symptoms	
Dysmenorrhea (mean ± SD)	6.49 ± 3.26
Chronic pelvic pain (mean ± SD)	2.94 ± 3.45
Dyspareunia (mean ± SD)	3.03 ± 3.36
Dysuria (mean ± SD)	0.67 ± 2.05
Dyschezia (mean ± SD)	2.02 ± 3.31
Abnormal uterine bleeding (*N* [%])	49 (22.6%)

**Table 2:** Demographic and clinical characteristics in the different patient groups.

Variables	DIE + adenomyosis (92)	Ovarian + adenomyosis (73)	Adenomyosis alone (52)	*P* value
Age (y) (mean ± SD)	34.4 ± 5.34	34.7 ± 6.36	37.8 ± 5.18	*P* = 0.021
BMI	22.6 ± 3.87	23.2 ± 3.81	23.0 ± 2.54	*P* = 0.541
Parity (*N* [%])				
0	87 (94.6%)	54 (73.9%)	27 (51.9%)	*P* < 0.0001
≥1	5 (5.4%)	19 (26.1%)	25 (48.1%)	*P* < 0.0001
History of previous surgery (≥1)	26 (28.3%)	26 (35.6%)	5 (9.6%)	*P* = 0.004
Symptoms				
Dysmenorrhea (mean ± SD)	7.04 ± 3.47	6.00 ± 3.46	6.21 ± 2.39	*P* = 0.096
Chronic pelvic pain (mean ± SD)	3.79 ± 3.58	2.83 ± 3.55	1.59 ± 2.54	*P* = 0.001
Dyspareunia (mean ± SD)	3.47 ± 3.54	3.35 ± 3.64	1.84 ± 2.15	*P* = 0.013
Dysuria (mean ± SD)	1.84 ± 2.15	0.50 ± 1.70	1.18 ± 2.68	*P* = 0.002
Dyschezia (mean ± SD)	3.32 ± 3.86	1.83 ± 2.98	0.01 ± 0.01	*P* < 0.0001
Abnormal uterine bleeding (*N* [%])	5 (5.4%)	23 (31.5%)	21 (40.4%)	*P* < 0.0001
